# Special issue: Metamaterials and plasmonics in Asia, a tribute to Byoungho Lee

**DOI:** 10.1515/nanoph-2023-0343

**Published:** 2023-06-22

**Authors:** Q-Han Park, Lei Zhou, Teruya Ishihara, Jeong Weon Wu

**Affiliations:** Korea University, Seoul, South Korea; Fudan University, Shanghai, China; Tohoku University, Sendai, Japan; Ewha Womans University, Seoul, South Korea

This special issue is a development of the 6th A3 Metamaterials Forum held from June 27 to 29, 2022, at Seoul National University, Korea. Namkyoo Park, from Seoul National University, served as the conference chair on behalf of Byoungho Lee due to his health issue at that time. Unfortunately, Byoungho Lee passed away on November 7, 2022, which was acknowledged by obituaries in the Nature Photonics journal [1] and the societies Optica [2] and SPIE [3]. His dedication to the *A3 Metamaterials Forum* (2016–2022) and the *Korea-Japan Metamaterials Forum* (2011–2015) contributed to maintaining the high quality of invited talks. Byoungho Lee himself was a top researcher in nanophotonics (plasmonics and metamaterials), holography, and 3D display. He left us with a legacy of relentless passion for scientific research and devoted leadership in professional societies such as the Optical Society of Korea, Optica, and SPIE. It is with deep respect that we dedicate this special issue to Byoungho Lee with a memorandum [4].

The capability to design and fabricate sub-wavelength artificial structures as inclusions has led to research on metamaterials with tailored effective-medium properties operating within a frequency range from microwave and terahertz to the visible spectral region, depending on the size of sub-wavelength inclusion. This special issue presents four review articles and 30 research articles.

The ease of implementing artificial gain and loss in optical processes renders nanophotonics a straightforward platform for exploring non-Hermitian physics in artificial optical structures. In addition, the concept of a topological insulator in condensed matter physics can be extended to optics platforms for a variety of advantages in optical device applications, the presence of a robust topological edge state being one prominent example. The advances and application of non-Hermitian topological photonics are elegantly presented in ref. [5], while ref. [6] covers the construction and manipulation of photonic topological states, and touches upon non-Hermitian topology. A metasurface is a two-dimensional metamaterial, where the Huygens wavefront of the outgoing wave is controlled by manipulating the amplitude, phase, and polarization of the incoming wave. This topic is extensively reviewed in ref. [7]. Additionally, ref. [8] reviews microscopic tools for super-resolution, fast volumetric imaging, and a large imaging field of view.

The emergence of exceptional points is characteristic of a non-Hermitian system, which can be extended to a photonic crystal structure with negative index media to realize a 2D symmetry-protected exceptional ring as shown in ref. [9].

Research articles on metasurfaces cover applications of angle-multiplexing in ref. [10], multilayer metalenses in ref. [11], high numerical aperture metalenses in ref. [12], anomalous diffraction in ref. [13], spatial filters in ref. [14], and bound-state-in-continuum based on nanohole array in ref. [15].

Active beam control is examined in schemes of deep-learning-assisted reconfigurable metasurface antenna in ref. [16], on-chip integration for optical phase array in ref. [17], and a digital and programmable metasurface in ref. [18].

An important feature of metamaterials is the versatility of energy-momentum dispersion engineering for broadband absorption, as well as epsilon-near-zero response. This is explored in a broadband absorber covering the entire microwave X-band in ref. [19], in cermet films with a nano-cone structure for a perfect absorber in ref. [20], in an all-dielectric broadband absorber in ref. [21], and in directive emission from highly-dispersive organics in ref. [22].

Nanoplasmonics is incorporated in various applications, such as the nanoimprint meta-device in ref. [23], the super-resolution localization microscope in ref. [24], the generation of a nano-vortex field associated with Laguerre-Gaussian beam in ref. [25], and plasmonic nanoantennas in ref. [26].

THz metamaterials and their applications are becoming increasingly important and are explored in various research articles. For instance, ultrafast time resolution nanoscale strong-field THz–matter interaction in ref. [27], wide reflection steering in the millimeter-wave and THz band in ref. [28], an isotropic metasurface suitable for planar optical components in 6G wireless communications in ref. [29], a THz optical nanofuse in ref. [30], and a THz metasurface wave retarder in ref. [31].

Polariton excitations, a coherent coupling of polarization and photons, are investigated in 2D structures for guided exciton-polariton in ref. [32] and polariton optics nanostructure-integrated low-dimensional devices in ref. [33]. Scattering is a key process in optics and exhibits characteristic features of nanophotonic structures. As such, a deep neural network is developed to facilitate the inverse design is detailed in ref. [34] and the semi-analytical method developed for engineering isospectrality in multi-dimensional photonic systems is explored in ref. [35].

Research articles exploring novel structured materials include an integration of active semiconductor into the conventional passive waveguide is demonstrated for chip-scale active nanophotonic platforms in ref. [36], hypersonic 3D phononic crystals designed and fabricated by DNA origami in ref. [37], and a jungle gym-shaped dielectric unit cell introduced for carpet cloaking with 3D anistropy control in ref. [38].

This special issue provides a comprehensive overview of research activities conducted by leading scientists in the field of metamaterials and plasmonics in Korea, Japan, and China. We hope that you will enjoy reading it.
